# Expression profiles of SGK-1 and α-ENaC in minor salivary glands of subjects with xerostomia

**DOI:** 10.3389/fdmed.2025.1585554

**Published:** 2025-08-05

**Authors:** Mahmood S. Mozaffari, Rafik Abdelsayed

**Affiliations:** ^1^ Department of Oral Biology, The Dental College of Georgia, Augusta University, Augusta, GA, United States; ^2^ Oral Health and Diagnostic Sciences, The Dental College of Georgia, Augusta University, Augusta, GA, United States

**Keywords:** lower lip biopsy, salivary glands, serum and glucocorticoid-regulated kinase-1, epithelial sodium channel, xerostomia

## Abstract

**Introduction:**

Epithelial sodium channel (ENaC) is a major conduit for sodium transport across the cell membrane, and its activity is regulated by multiple factors/mechanisms, including the serum and glucocorticoid-regulated kinase-1 (SGK-1). Saliva production and secretion are complex processes, with ENaC regulation of the ionic composition of saliva being an essential event prior to the ultimate secretion of hypotonic saliva into the oral cavity. However, the status of salivary gland SGK-1, in the context of ENaC, remains to be determined. We tested the hypothesis that lower lip minor salivary gland expressions of SGK-1 and ENaC are affected in subjects reporting xerostomia.

**Methods:**

Accordingly, archived biopsy specimens of subjects with a diagnosis of mucocele (control; *n* = 7) and those of subjects complaining of dry mouth (experimental; *n* = 12) were subjected to histopathological and immunohistochemical assessments for SGK-1, its phosphorylated (active) form (pSGK-1), and the alpha subunit of ENaC (α-ENaC).

**Results:**

Control specimens displayed extravasated mucus surrounded by a capsule of inflamed granulation tissue, while experimental specimens showed patchy periductal, predominantly lymphocytic, infiltrates. Control specimens showed variable degrees of SGK-1 and pSGK-1 immunolabeling of ductal epithelial cells. In contrast, experimental specimens displayed patchy and strong SGK-1 but variable degrees of pSGK-1 immunolabeling of ductal epithelial cells. While control specimens showed variable ductal α-ENaC immunolabeling, those of the experimental group displayed primarily diffuse cytoplasmic, with some membrane, immunolabeling in ductal cells. Semi-quantitative analyses, using ImageJ Fiji, showed increased normalized staining for α-ENaC and SGK-1, but not pSGK-1, for experimental compared to control cases.

**Discussion:**

Collectively, the data suggest a difference between the active form of the kinase and α-ENaC in minor salivary glands in xerostomia and that higher SGK-1 and α-ENaC may serve as diagnostic markers for this condition.

## Introduction

Saliva has multifaceted functions in oral soft and hard tissues that are essential for oral health maintenance. These include hydration and lubrication of the oral cavity, participation in food digestion, clearance of substances, buffering of pH, maintenance of tooth mineralization, antimicrobial effects, and promotion of wound healing. While xerostomia is the subjective reporting of oral dryness, given the multiple effects of saliva, salivary hypofunction is associated with major adverse outcomes, including dry, pale, and atrophic soft tissues; a fissured and inflamed tongue; propensity for dental caries; and opportunistic infections (1).

Whole saliva is the composite of the secretions from the major and minor salivary glands. Acinar cells secrete an isotonic fluid, the ionic composition of which is determined by the polarity of acinar cells, given the presence of different ion transport proteins on apical and basolateral cell membranes, apical water channels, and movement of water and sodium via leaky tight junctions. As saliva moves from the acinar lumen through the ductal system, it becomes hypotonic because of the coordinated actions of various ion transport proteins and a lack of water channels or leakiness of tight junctions (1, 2).

The epithelial sodium channel (ENaC) on the apical membrane of ductal cells is an important determinant of the production of hypotonic saliva. ENaC is constitutively expressed and is present mostly in tight or high-resistance epithelium. It is a conduit for apical transport of sodium from the lumen into epithelial cells, which, in turn, is removed from the cell into the interstitial fluid by basolateral Na^+^/K^+^ ATPase (2, 3). Since the ENaC is a major determinant of the sodium concentration of extracellular fluid, its roles in renal tubular reabsorption of sodium, body fluid homeostasis, and blood pressure regulation have been the focus of intense investigation (3). Thus, aside from well-known regulators of ENaC (e.g., renin-angiotensin-aldosterone axis), a complex array of extracellular factors (e.g., Na^+^, Cl^−^, H^+^), shear stress, proteases, and kinases reportedly regulate ENaC activity (4). Among kinases, serum and glucocorticoid-regulated kinase-1 (SGK-1), the expression of which is regulated by aldosterone (5), has received considerable attention. For example, the association of SGK-1 with the apical membrane and its interaction with the ENaC increases channel activity in aldosterone-stimulated renal collecting duct cells (6). Using the A6 cell line (a *Xenopus laevis* cell line isolated from the kidney), additional impacts of SGK-1 on ENaC have been proposed, including increased probability of channel opening and activation of pre-existing channels within apical cell membranes and/or intracellular compartments (7). Thus, it is likely that SGK-1 regulates salivary gland ENaC expression and/or activity, aspects that remain to be elucidated.

In light of the above, we sought to determine the status of SGK-1 and ENaC in archived lower lip biopsy samples from patients with a diagnosis of mucocele (control cases) and patients who had presented with a chief complaint of xerostomia; histopathological and immunohistochemical assessments of minor salivary gland biopsy specimens are believed to be valuable in classification and diagnosis of salivary gland pathologies and systemic manifestations (8). We tested the hypothesis that expressions of SGK-1 and ENaC are affected in the lower lip minor salivary gland specimens from patients reporting xerostomia.

## Materials and methods

The archived tissue bank of the diagnostic pathology laboratory at the Dental College of Georgia was searched for cases with diagnosis of mucocele (control cases; *n* = 7) and those of subjects with a chief complaint of xerostomia (*n* = 12) for whom a tissue biopsy was obtained; sample sizes were based on our recent experience using archived human biopsy specimens (9). This retrospective study was considered exempt from review by the Institutional Review Board. These cases were initially assessed by relying on clinical presentation and histopathological features of biopsy specimens using hematoxylin–eosin (H&E) stain.

The corresponding paraffin-embedded tissue blocks of the aforementioned cases were identified, 5 μm tissue sections were deparaffinized in a Leica Auto-Stainer XL (Leica Biosystems, Nussloch, Germany), and antigen retrieval was performed using a citric acid-based antigen unmasking solution (Vector Laboratories, Burlingame, CA, USA). Thereafter, tissue sections were treated with 0.3% hydrogen peroxide for 30 min at room temperature, washed in water, and then incubated with blocking solution [2.5% horse serum, 1% bovine serum albumin (BSA), 0.5% Triton X-100] for at least 1 h at room temperature. Each primary antibody was diluted, as indicated below, in blocking solution and incubated with tissue sections overnight at room temperature. SGK-1 rabbit polyclonal antibody was obtained from ABclonal (catalog number A1025; 1;100 dilution), pSGK-1 rabbit polyclonal antibody (Ser422) was obtained from Invitrogen (catalog number 44-1264G; 1;100 dilution), and α-ENaC rabbit polyclonal antibody (BS-2957R; 1:100 dilution) was purchased from Thermo Fisher Scientific (MA, USA); the α subunit of ENaC is critical for ion channel activity and pSGK-1 is the active form of the kinase (9, 10). Tissue sections were then washed twice in phosphate buffered saline (PBS) and incubated for 1 h at room temperature with horseradish peroxidase-conjugated secondary antibody (horse anti-rabbit IgG polymer kit, peroxidase: MP-7401; Vector Laboratories, Burlingame, CA, USA) followed by 3,3′-diaminobenzidine (DAB) staining using the ImmPACT DAB Substrate Kit (SK-4105; Vector Laboratories, Burlingame, CA, USA). Slides were counterstained with hematoxylin and mounted using mounting medium. As a positive control, human mammary tissue was used for SGK-1 and pSGK-1, while human kidney tissue was used for α-ENaC; negative controls excluded the primary antibody against each protein of interest (Supplementary Figure S1).

ImageJ Fiji software was used for the semi-quantitative analyses of the immunolabeling following a previously described detailed step-by-step protocol (9, 11, 12). This protocol involves deconvolution of immunohistochemistry images followed by assessment of DAB staining, using mean gray value, and normalization to the nuclei.

### Statistics

Semi-quantitative data are reported as mean ± SEM. Data for each protein of interest were analyzed using an unpaired Student's *t*-test to establish significance (*p* < 0.05) between control and experimental specimens.

## Results

### Subjects’ demographics

The cases used for this retrospective study were sent to the diagnostic pathology laboratory with relevant demographic information but no reference to the patient's medical history or medication profile. Tables 1 and 2 show demographic information, site of lesion, and clinical impression/diagnosis (i.e., prior to histopathological examination and immunolabeling). Briefly, the control subjects (71% Caucasians), four female and three male subjects, were 10–49 years of age, while all except one of the experimental subjects (75% Caucasians) were females and 36–70 years of age. The vast majority of experimental subjects were suspected of having Sjögren's syndrome (SS) at the time of their initial clinical assessment.

**Table 1:** Demographic information of the control subjects.

Subjects	Age (years)	Sex	Ethnicity	Anatomical site	Initial clinical impression/diagnosis
Subject 1	36	Female	Caucasian	Lower lip, right side	Mucocele, calcified minor salivary gland, sialadenitis
Subject 2	18	Female	Caucasian	Lower lip, right side	Mucocele, traumatic fibroma
Subject 3	49	Male	Caucasian	Lower lip, left side	Mucocele
Subject 4	10	Male	African-American	Lower lip, left side	Mucocele
Subject 5	11	Male	African-American	Lower lip, midline area	Mucocele
Subject 6	18	Female	Caucasian	Lower lip, right side	Mucocele
Subject 7	33	Male	Caucasian	Lower lip, right side	Mucocele

**Table 2:** Demographic information of the experimental subjects.

Subjects	Age (years)	Sex	Ethnicity	Anatomical site	Initial clinical impression/diagnosis
Subject 1	48	Female	Hispanic	Lower lip	Suspected Sjogren’s syndrome
Subject 2	63	Female	Caucasian	Lower lip	Suspected Sjogren’s syndrome
Subject 3	54	Female	Caucasian	Lower lip	Recurrent aphthous ulcers
Subject 4	54	Female	Caucasian	Lower lip, right side	Suspected Sjogren's syndrome
Subject 5	36	Female	Caucasian	Lower lip, right side	Suspected Sjogren's syndrome
Subject 6	51	Female	Caucasian	Lower lip	Sjogren’s syndrome ruled out; granuloma
Subject 7	65	Male	Caucasian	Lower Lip	Sjogren's syndrome ruled out, traumatic candidal ulceration
Subject 8	52	Female	Caucasian	Lower lip	Suspected Sjogren's syndrome
Subject 9	47	Female	Hispanic	Left Parotid	Suspected Sjogren's syndrome
Subject 10	62	Female	Caucasian	Lower lip, left side	Probable mucocele (evaluated for Sjogren’s syndrome)
Subject 11	52	Female	African-American	Lower lip	Suspected Sjogren's syndrome
Subject 12	70	Female	Caucasian	Lower lip, right side	Xerostomia, Sjogren's syndrome ruled out

### Hematoxylin and eosin staining

**Figure 1:**
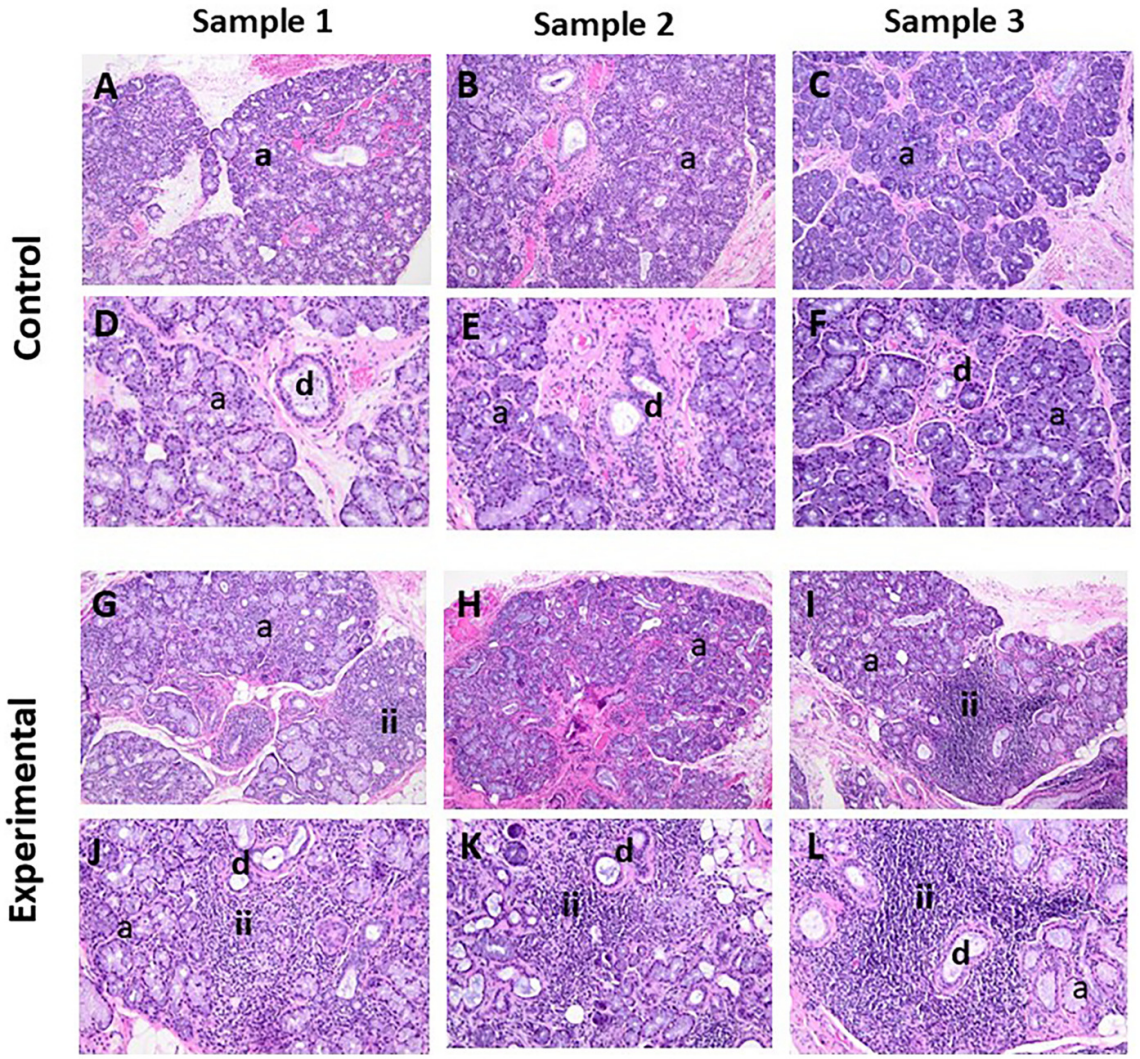
The panels show representative H&E images for control and experimental tissue samples. Compared to the control cases, the experimental specimens display marked periductal inflammatory infiltrates and associated destruction of acinar cells (i.e., acinar atrophy). **(A–C**, **G–I)** 10× and **(D–F**, **J–L)** 20×. a, acini; d, duct; ii, inflammatory infiltrates

The H&E-stained sections of control cases showed sections of labial salivary glands, some of which were covered by parakeratinized squamous epithelium. The most significant feature of these specimens was the presence of pools of extravasated mucus surrounded by a capsule of inflamed granulation tissue (Supplementary Figure S2). The extravasated mucus supports numerous swollen macrophages accompanied by variable numbers of neutrophils. The surrounding salivary glands exhibited the normal lobular architecture with occasional intralobular acinar atrophy, irregular ductal dilatation, and interstitial chronic inflammation, predominantly lymphocytes and plasma cells (Figures 1A–F). The surface mucosa is essentially within normal limits.

The H&E-stained sections of the experimental cases showed sections of labial salivary glands that maintained the normal lobular architecture (Figures 1G–L). The glands supported patchy periductal, predominantly lymphocytic, infiltrates. There was no significant interstitial fibrosis or mucus extravasation noted, but there were variable degrees of acinar atrophy (due to destruction of acinar cells), particularly in the periductal areas where lymphocytic infiltrations were noted. In most cases, neurovascular bundles were evident.

### Immunohistochemistry

#### SGK-1 and pSGK-1

**Figure 2:**
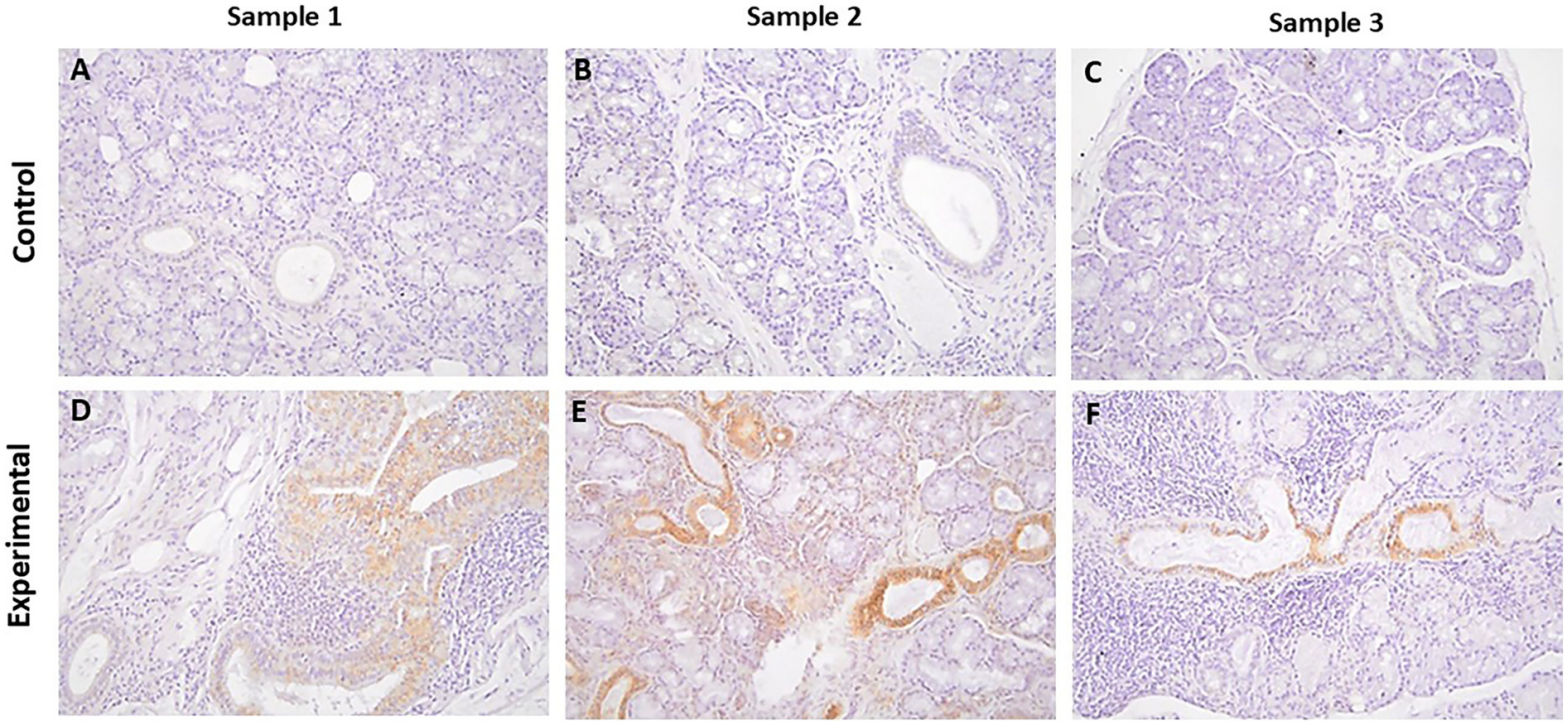
The panels show representative images for SGK-1 for control and experimental tissue samples. Compared to the control specimens, experimental cases display prominent immunolabeling in ductal cells. **(A–F)** 20×.

**Figure 3:**
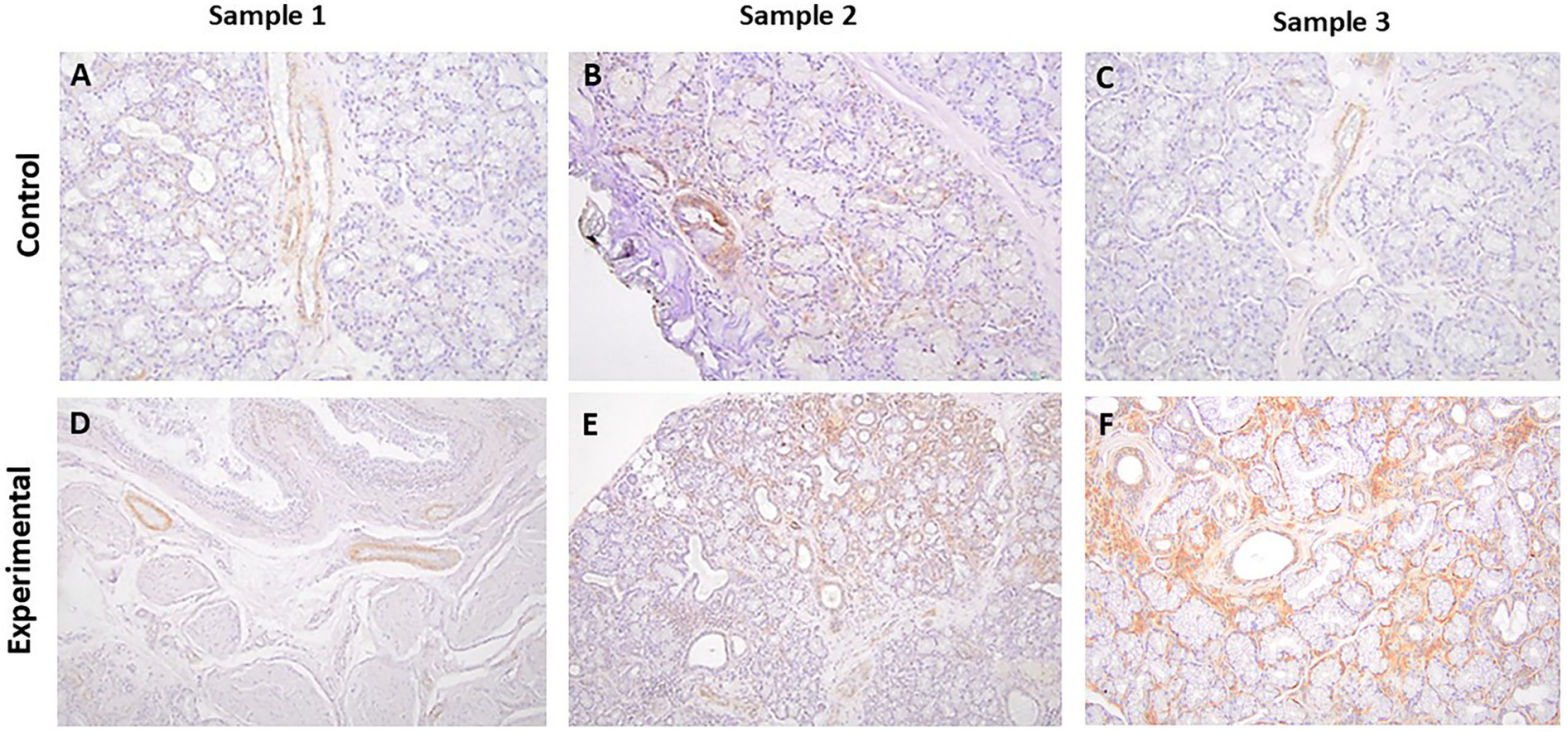
The panels show representative images for pSGK-1 for control and experimental tissue samples. The control and experimental specimens display variable degrees of immunolabeling in ductal structures. **(A–F)** 20×.

The tissue sections of control specimens subjected to immunochemistry for SGK-1 (Figures 2A–C) or pSGK-1 (Figures 3A–C) showed variable degrees of immunolabeling intensity of the ductal epithelial cells. Tissue sections of experimental cases stained with SGK-1 showed a patchy distribution with strong staining intensity of the ductal epithelial cells (Figures 2D–F). Further, experimental tissue sections stained for pSGK-1 showed variable degrees of staining intensity of the ductal epithelial cells (Figures 3D–F).

It is noteworthy that the control specimens did not show appreciable SGK-1 or pSGK-1 immunolabeling in acinar units, stromal cells, extravasated mucus, or surrounding granulation tissue; however, occasional immunolabeling for peri-acinar myoepithelial cells and vascular smooth muscles was noted in some cases. Similarly, the experimental specimens did not exhibit immunolabeling for SGK-1 or pSGK-1 of acinar units, stromal cells, or periductal lymphocytes. However, in some experimental sections, immunolabeling was noted for non-ductal cells such as myoepithelial and vascular smooth muscle cells (Supplementary Figure S3).

#### α-ENaC

**Figure 4:**
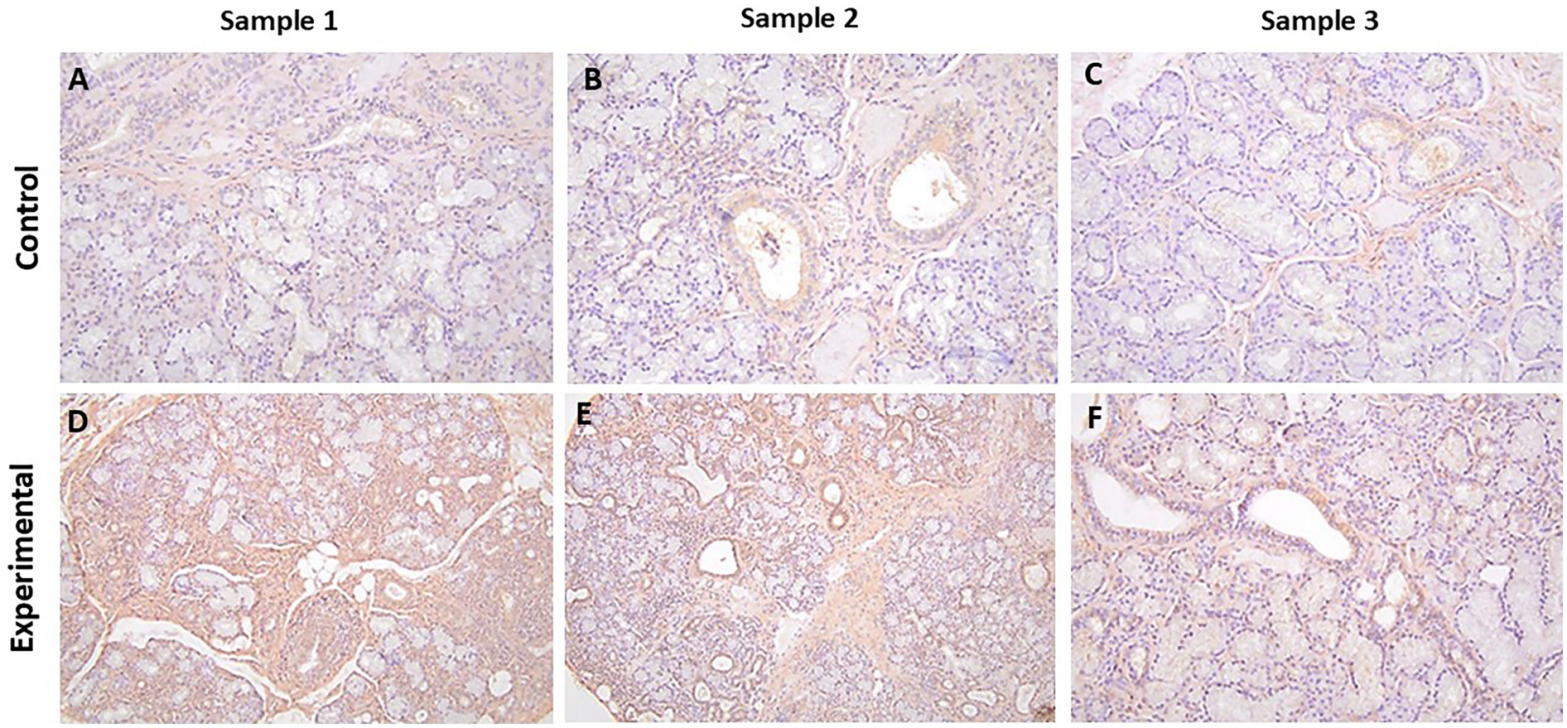
The panels show representative images for α-ENaC for control and experimental tissue samples. The control cases showed variable degrees of immunolabeling, while the experimental specimens displayed more prominent immunolabeling in ductal cells. **(A–F)**) 20×.

The tissue sections of control specimens stained with α-ENaC showed prominent membrane immunolabeling in ductal cells, with occasional positive myoepithelial cells and a positive reaction with stromal cells (Figures 4A–C, Supplementary Figure S4).

The tissue sections of experimental cases stained for α-ENaC showed primarily diffuse cytoplasmic, with some membrane, immunolabeling in ductal cells with positive myoepithelial cells and a positive reaction with stromal cells (Figures 4D–F, Supplementary Figure S4). None of the cases showed a positive reaction in the periductal lymphocytes.

### Semi-quantitative analyses

**Figure 5:**
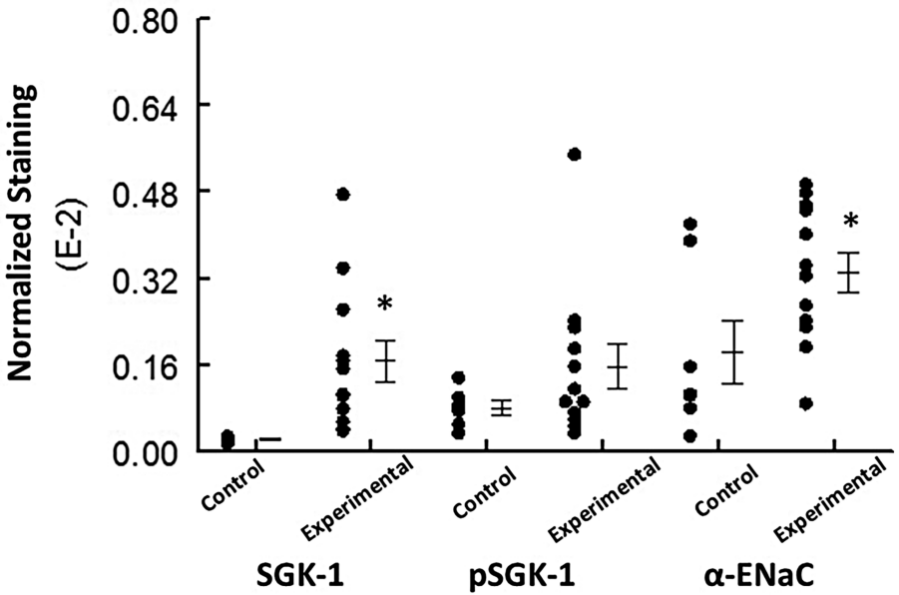
The graph shows individual values and mean ± SEM for normalized immunolabeling for the proteins of interest in the control and experimental groups. *p < 0.05 compared to the experimental group for the same protein.

The experimental group displayed significantly (*p* < 0.05) greater normalized staining for SGK-1 and α-ENaC compared to the control group; however, this parameter was similar for pSGK-1 between the two groups (Figure 5).

## Discussion

The present study showed increased expression of α-ENaC and SGK-1, but not pSGK-1, in minor salivary glands of subjects reporting xerostomia compared to those of control subjects with presentation of mucocele. These novel observations suggest a difference between α-ENaC and the active form of SGK-1 in xerostomia.

The ENaC is expressed in epithelial and non-epithelial cells of various organs and tissues throughout the body, including the kidney, colon, lung, and reproductive organs, among others. Recent studies indicate that the human ENaC is a heterotrimer of homologous subunits of *α*, *β,* and *γ* with a stoichiometry of 1:1:1 (13). However, a fourth subunit, *δ*, has been reported in some tissues (e.g., vessels, heart, and lungs) that can substitute for the *α* subunit and confer different functions, such as salt and sour taste sensation, mechanosensation, and sperm motility, among others (3, 4, 14). Importantly, independent expression of ENaC subunits in *X. laevis* oocytes or rat thyroid epithelial cells revealed that only the *α* subunit actively transports sodium and that its coexpression with *β* and *γ* subunits increases the sodium current (15, 16). The significance of the ENaC to human biology is exemplified by disease conditions (e.g., Liddle syndrome), whereby mutations in the ENaC result in its dysfunction and increased renal expression/activity and consequent augmented sodium and fluid reabsorption, causing salt-sensitive hypertension (17–19). With respect to salivary glands, the rat submandibular epithelial cell line displayed increased ENaC expression and basal epithelial sodium transport in response to hydrocortisone and aldosterone (10). Further, ligation of submandibular gland ducts in rats increased expression of ENaC subunits, suggesting a pathogenic role in obstructive sialadenitis (20). A recent study explored salivary sodium composition in the context of salivary gland ENaC expression in subjects with primary SS (21). The results showed increased saliva sodium concentration under both stimulated and non-stimulated conditions in subjects with SS compared with non-SS sicca controls. Further, parotid gland tissue from non-SS sicca controls displayed apical expression of ENaC on the luminal surface of striated ducts, but this pattern was absent in parotid gland tissue of subjects with SS. The authors concluded that the pro-inflammatory environment in SS may dysregulate the ENaC (21). Our results indicate increased α-ENaC expression in the minor salivary gland of subjects reporting xerostomia in association with intense periductal infiltrates, primarily lymphocytes. Interestingly, however, α-ENaC expression was primarily cytoplasmic rather than being present on the apical membrane of ductal cells, the latter being a requirement for functional channel activity. Collectively, these observations are suggestive of dysregulation of the ENaC in the minor salivary glands of the experimental subjects.

Multiple factors and mechanisms regulate the ENaC, with the role of SGK-1 being well-characterized. For example, in aldosterone-stimulated collecting duct cells, SGK-1 is reportedly active on the apical membrane, and its interaction with the ENaC increases sodium channel activity (6); ENaC activity constitutes the rate-limiting step in sodium reabsorption and thereby sodium balance and blood pressure regulation. Further, in the A6 renal epithelial cell line with constitutive expression of SGK-1, the effects of SGK-1 include increased probability of ENaC channel opening, increased abundance of ENaC subunits within apical membranes and intracellular compartments, and activation of one or more pools of pre-existing channels within the apical membranes and/or intracellular compartments (7). The stimulatory effect of SGK on ENaC may be related to the phosphorylation of ubiquitin protein ligase, Nedd4-2, thereby preventing its inhibition of the channel activity and its removal from the membrane (22). Additional mechanisms for SGK-1 regulation of the ENaC include transcription of ENaC subunits and increasing ENaC activity directly via phosphorylation of its subunits (23). We found increased expression of SGK-1, but not pSGK-1, in the minor salivary glands of subjects reporting xerostomia than in control subjects. Since the pSGK-1 is the active form of SGK-1, it is likely that the primarily ductal cell cytoplasmic expression of α-ENaC in the experimental specimens relates to dysregulation of ENaC trafficking between apical membranes and intracellular compartments.

For this retrospective study, we relied on the use of archived biopsy specimens that were previously sent to our diagnostic pathology laboratory for histopathological evaluation. Thus, we do not have access to relevant patient information such as medical history, medication profile, laboratory results, saliva production, and the ultimate clinical diagnosis. Nonetheless, the presence of intense periductal lymphocytic infiltrates, along with the destruction of acini, was a characteristic feature of the tissue specimens from the subjects (92% female) reporting xerostomia who, at the time of their initial clinical assessment, were suspected of having Sjögren's syndrome. Given that a firm diagnosis of this condition is multifaceted (24), and despite our histopathological findings, we have referred to experimental subjects based on their chief complaint of xerostomia. Nonetheless, it is likely that the inflammatory microenvironment and consequent adverse impact on salivary gland function contributed to the subject's chief complaint of dry mouth.

Although dry mouth is a hallmark feature of Sjögren’s syndrome, the condition is associated with other general and immune system-related comorbidities (24). Thus, aside from the use of serological biomarkers and histopathology of the minor salivary glands for diagnosis, intense research has focused on the identification of molecular biomarkers. While many potential candidates have been investigated, they have not evolved into validated diagnostic tools. Nonetheless, a number of molecules have received considerable attention as potential novel biomarkers, including calprotectin, carbamylated proteins (e.g., homocitrulline), antibodies targeting muscarinic subtype 3 receptors, β2 microglobulin, and neutrophil gelatinase-associated lipocalin, among others (24, 25). In this retrospective study of subjects with xerostomia (and suspected of having Sjögren’s syndrome), we observed increased expressions of SGK-1 and α-ENaC, raising the question of their utility as diagnostic biomarkers for this condition. Clearly, however, extensive pre-clinical and clinical studies are needed to establish a cause-and-effect relationship between their expression and salivary gland function, as well as validation studies linking their altered expression to salivary hypofunction.

In conclusion, despite a relatively small sample size, the present study showed increased expression of SGK-1 and α-ENaC in the ductal cells of the minor salivary glands of subjects reporting xerostomia compared to control subjects. Nonetheless, pSGK-1 (i.e., active kinase) was similar between the control and experimental groups, likely accounting for primarily cytoplasmic α-ENaC immunolabeling given the role of this kinase in its cellular trafficking (18, 22, 23). Since apical membrane localization of α-ENaC is required for functional ENaC activity (3, 5–6, 18), it is likely that, despite its increased expression in the biopsy specimens from experimental subjects, SGK-1-mediated regulation of ENaC function is impaired in conditions associated with salivary gland dysfunction.

## Data Availability

The raw data supporting the conclusions of this article will be made available by the authors, without undue reservation.
