# Supplementary material for: Expression profiles of SGK-1 and α-ENaC in minor salivary glands of subjects with xerostomia
Source: Front Dent Med. 2025 Aug 5;6:1585554. doi: 10.3389/fdmed.2025.1585554 (PMC12361188; doi:10.3389/fdmed.2025.1585554)
Supplement: Supplementary file 1 [file Presentation1.pptx]

## Slide 1
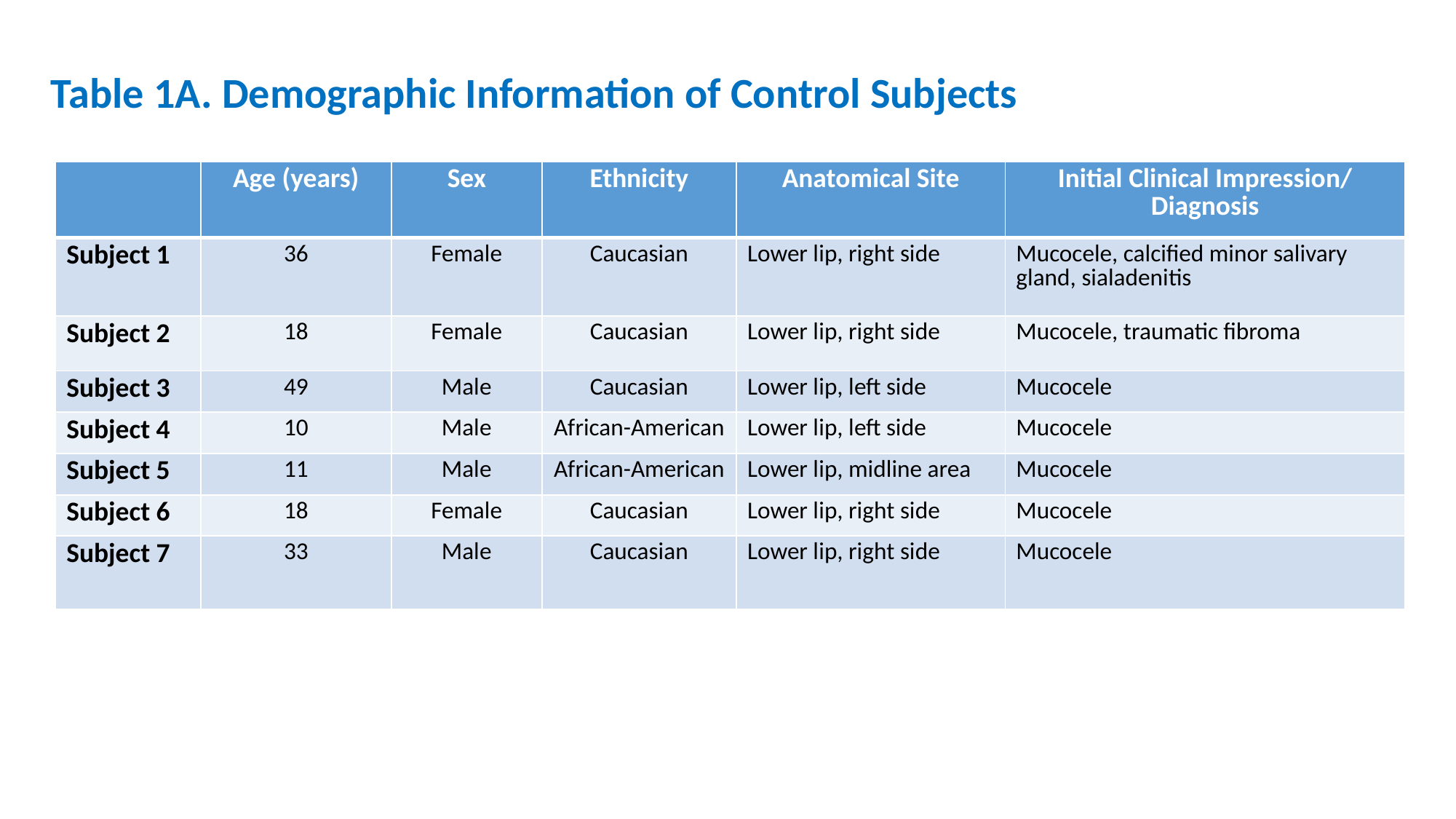

Table 1A. Demographic Information of Control Subjects
| | Age (years) | Sex | Ethnicity | Anatomical Site | Initial Clinical Impression/ Diagnosis |
| --- | --- | --- | --- | --- | --- |
| Subject 1 | 36 | Female | Caucasian | Lower lip, right side | Mucocele, calcified minor salivary gland, sialadenitis |
| Subject 2 | 18 | Female | Caucasian | Lower lip, right side | Mucocele, traumatic fibroma |
| Subject 3 | 49 | Male | Caucasian | Lower lip, left side | Mucocele |
| Subject 4 | 10 | Male | African-American | Lower lip, left side | Mucocele |
| Subject 5 | 11 | Male | African-American | Lower lip, midline area | Mucocele |
| Subject 6 | 18 | Female | Caucasian | Lower lip, right side | Mucocele |
| Subject 7 | 33 | Male | Caucasian | Lower lip, right side | Mucocele |

## Slide 2
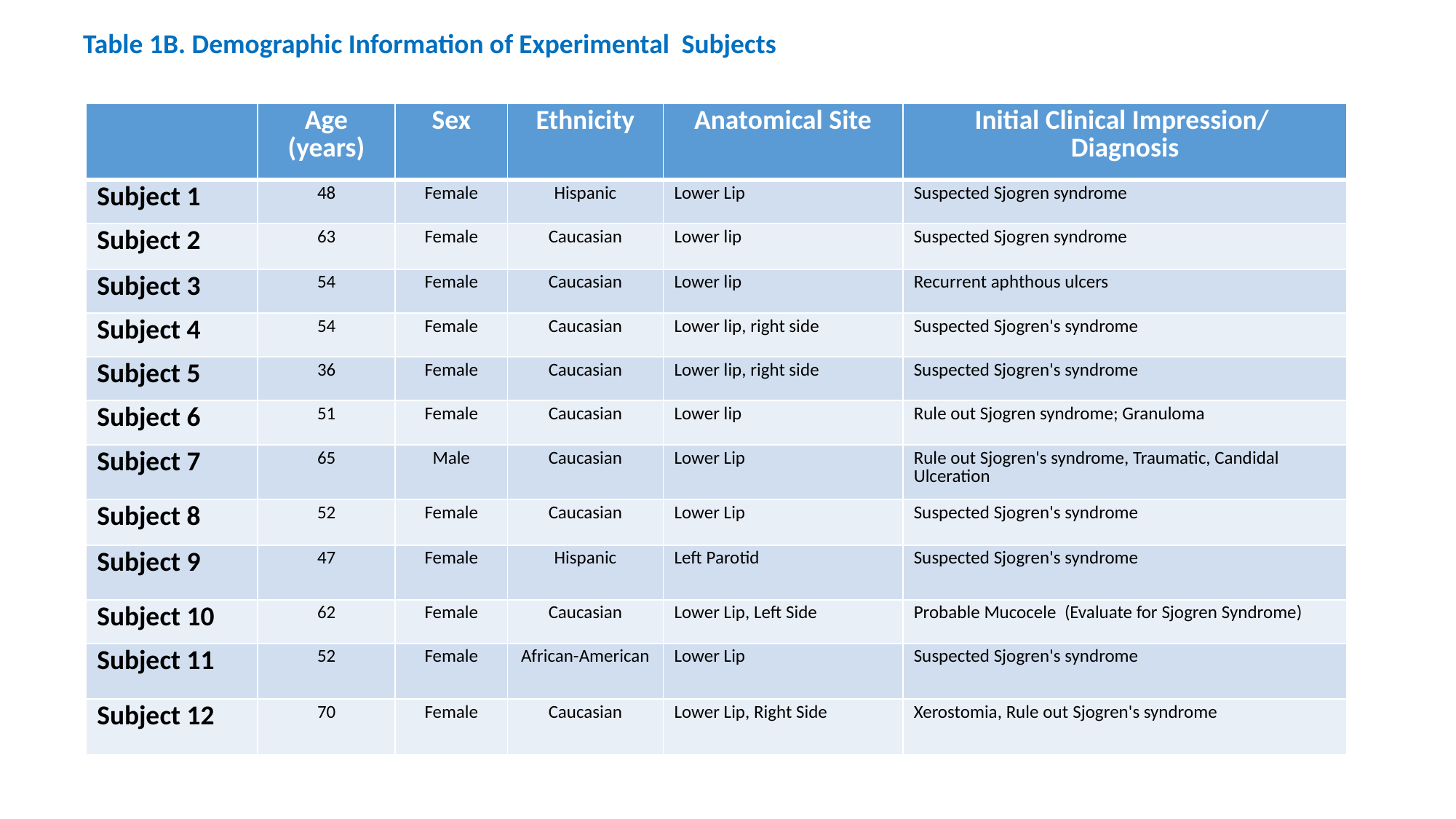

Table 1B. Demographic Information of Experimental Subjects
| | Age (years) | Sex | Ethnicity | Anatomical Site | Initial Clinical Impression/ Diagnosis |
| --- | --- | --- | --- | --- | --- |
| Subject 1 | 48 | Female | Hispanic | Lower Lip | Suspected Sjogren syndrome |
| Subject 2 | 63 | Female | Caucasian | Lower lip | Suspected Sjogren syndrome |
| Subject 3 | 54 | Female | Caucasian | Lower lip | Recurrent aphthous ulcers |
| Subject 4 | 54 | Female | Caucasian | Lower lip, right side | Suspected Sjogren's syndrome |
| Subject 5 | 36 | Female | Caucasian | Lower lip, right side | Suspected Sjogren's syndrome |
| Subject 6 | 51 | Female | Caucasian | Lower lip | Rule out Sjogren syndrome; Granuloma |
| Subject 7 | 65 | Male | Caucasian | Lower Lip | Rule out Sjogren's syndrome, Traumatic, Candidal Ulceration |
| Subject 8 | 52 | Female | Caucasian | Lower Lip | Suspected Sjogren's syndrome |
| Subject 9 | 47 | Female | Hispanic | Left Parotid | Suspected Sjogren's syndrome |
| Subject 10 | 62 | Female | Caucasian | Lower Lip, Left Side | Probable Mucocele (Evaluate for Sjogren Syndrome) |
| Subject 11 | 52 | Female | African-American | Lower Lip | Suspected Sjogren's syndrome |
| Subject 12 | 70 | Female | Caucasian | Lower Lip, Right Side | Xerostomia, Rule out Sjogren's syndrome |
