# Supplementary material for: Expression profiles of SGK-1 and α-ENaC in minor salivary glands of subjects with xerostomia
Source: Front Dent Med. 2025 Aug 5;6:1585554. doi: 10.3389/fdmed.2025.1585554 (PMC12361188; doi:10.3389/fdmed.2025.1585554)
Supplement: Supplementary file 2 [file Datasheet2.pdf]

Supplemental File

Legends for Supplemental Figures:

Supplemental Figure 1: Images show immunolabeling for SGK-1 (A) and pSGK-1 (B) in mammary tissue as well as α-ENaC (C) in the kidney. Panels D-F show corresponding images for the same tissue, protein and protocol but without the use of the primary antibody. 20x: A-F

Supplemental Figure 2: H&E images of two cases of mucocele show the appearance of extravasated mucin surrounded by a capsule of inflamed granulation tissue. The surface epithelium appears generally intact. e: Epithelium; gt: Granulation Tissue; m: Mucin; ii: Inflammatory Infiltrates. 4x: A-B; 10x: C-D

Supplemental Figure 3: Panels A and B show immunolabeling of myoepithelial cells for SGK-1 (control specimen) and pSGK-1 (experimental specimen), respectively. Panel C shows immunolabeling of a blood vessel for pSGK-1 of an experimental specimen. 40x: A-B; 20x: C

Supplemental Figure 4: Images of control and experimental minor salivary glands (A and B, respectively) show more marked apical expression of α-ENaC (black arrows) in the control specimen. Red arrows show α-ENaC expression in myoepithelial cells. 20x: A-B.

Supplemental Figure 1:

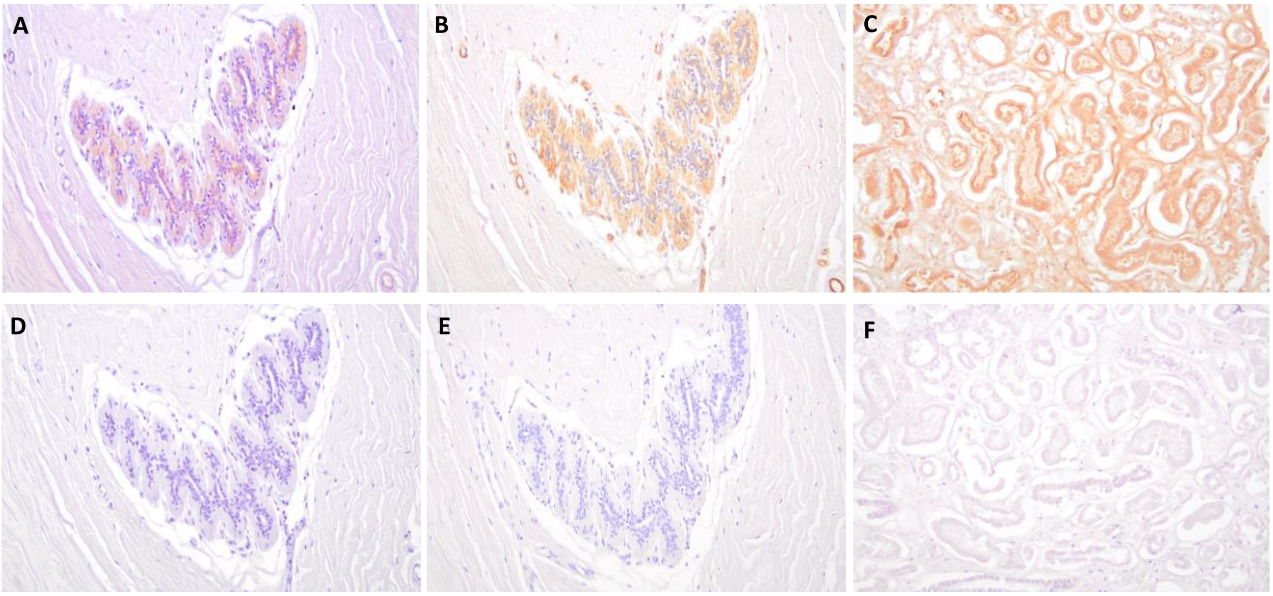

Supplemental Figure 1.
Mozaffari & Abdelsayed 2025

Supplemental Figure 2:

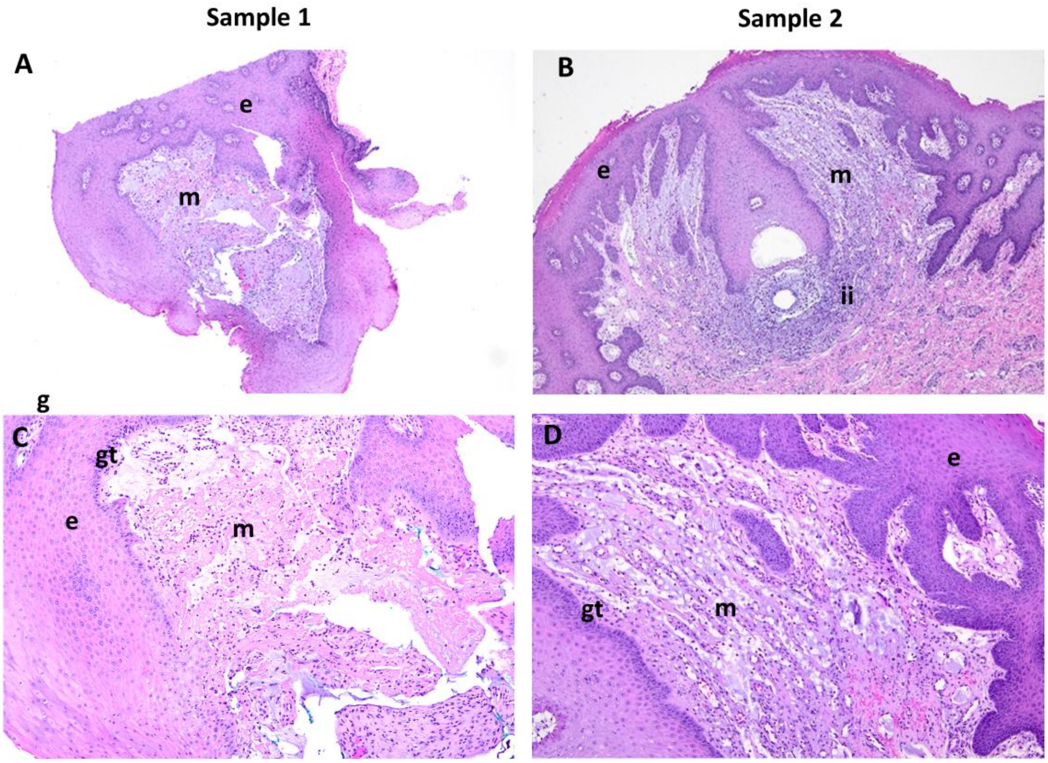

Supplemental Figure 2.
Mozaffari & Abdelsayed 2025

Supplemental Figure 3:

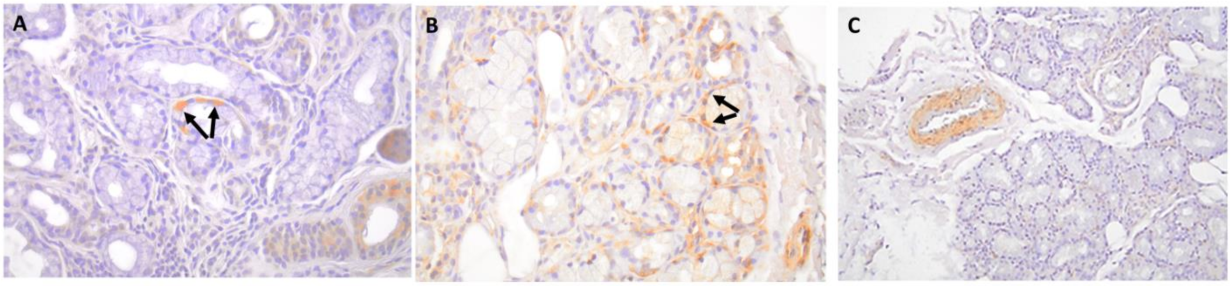

Supplemental Figure 3.
Mozaffari & Abdelsayed 2025

Supplemental Figure 4:

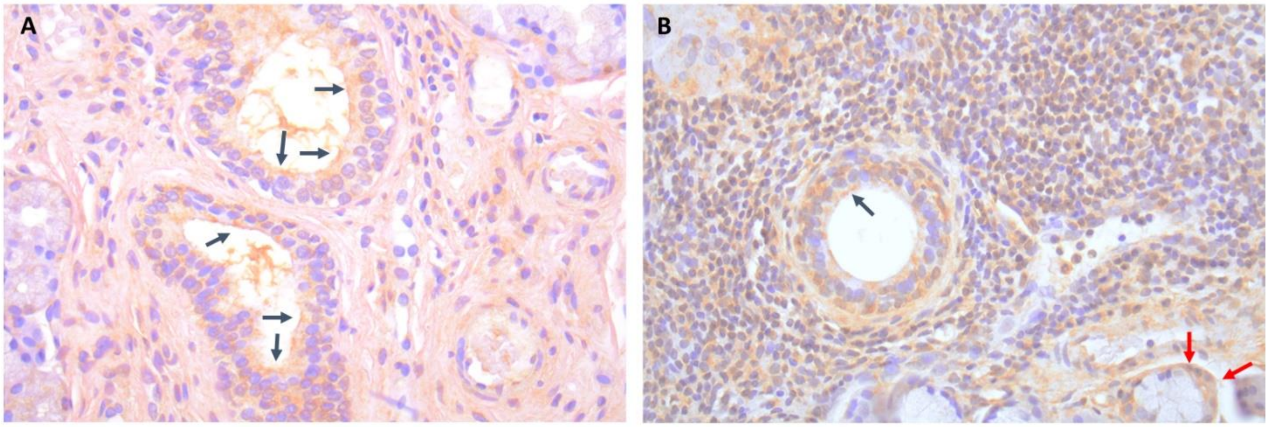

Supplemental Figure 4.
Mozaffari & Abdelsayed 2025
